# CD36 tango in cancer: signaling pathways and functions

**DOI:** 10.7150/thno.36037

**Published:** 2019-07-09

**Authors:** Jingchun Wang, Yongsheng Li

**Affiliations:** Clinical Medicine Research Center, Xinqiao Hospital, Army Medical University, Chongqing 400037, China.

**Keywords:** CD36, cancer treatment, biomarker, lipid metabolism, tumor-associated immune cell

## Abstract

CD36, a scavenger receptor expressed in multiple cell types, mediates lipid uptake, immunological recognition, inflammation, molecular adhesion, and apoptosis. CD36 is a transmembrane glycoprotein that contains several posttranslational modification sites and binds to diverse ligands, including apoptotic cells, thrombospondin-1 (TSP-1), and fatty acids (FAs). Beyond fueling tumor metastasis and therapy resistance by enhancing lipid uptake and FA oxidation, CD36 attenuates angiogenesis by binding to TSP-1 and thereby inducing apoptosis or blocking the vascular endothelial growth factor receptor 2 pathway in tumor microvascular endothelial cells. Moreover, CD36-driven lipid metabolic reprogramming and functions in tumor-associated immune cells lead to tumor immune tolerance and cancer development. Notable advances have been made in demonstrating the regulatory networks that govern distinct physiological properties of CD36, and this has identified targeting CD36 as a potential strategy for cancer treatment. Here, we provide an overview on the structure, regulation, ligands, functions, and clinical trials of CD36 in cancer.

## Introduction

Lipid metabolism is currently attracting increasing research attention because of its critical role in tumor initiation, development, and metastasis 1. Adipocytes supply adequate fatty acids (FAs) to meet the high energy requirement of tumor cells and fuel their development, and tumor cells also show increased propensity to migrate toward adipose tissues, where they can uptake sufficient lipids 2. CD36, a transmembrane glycoprotein (GP) that is also known as FA translocase (FAT), platelet GPIV, GP88, and scavenger receptor class B type 2 (SR-B2), is expressed on the cell surface in multiple cell types, including dendritic cells (DCs), microvascular endothelial cells (MVECs), retinal epithelial cells, monocytes, adipocytes, platelets, enterocytes, microglial cells, and podocytes 3. In tumor tissues, CD36 is expressed in tumor cells and in stromal and immune cells, but the expression level varies in distinct cell types and tumor stages. CD36 expression is invariably low in the in situ stage but increases during metastasis 4, and CD36 plays essential roles in lipid homeostasis, angiogenesis, immune response, adhesion, and metastasis in cancer 4, 5. Therefore, CD36 is now regarded as a potential biomarker and therapeutic target for cancer.

## CD36: discovery, structure, and distribution

### Discovery of CD36

In 1973, Kobylka and Carraway discovered a membrane protein in breast epithelial cells that could not be hydrolyzed in milk fat globules 6, and in 1978, the molecule was identified as platelet GPIV, a protein that mediates thrombospondin-1 (TSP-1) binding in platelets 7. Subsequently, Tandon found that GPIV featured an overlapping structure with leukocyte-differentiation antigen CD36, and other investigators later determined the effects of CD36 in platelet activation 8 and cell adhesion 9. In 1993, Abumrad and colleagues demonstrated the FAT activity of CD36, after which the protein in mice was cloned and identified as the mouse ortholog of human CD36, which indicated its role in FA uptake 10. CD36 is now widely recognized as a scavenger receptor that can absorb long-chain FAs (LCFAs) and oxidized low-density lipoprotein (ox-LDL) 11, 12.

### Structure of CD36

The human CD36 gene is located at chromosome 7q11.2 and is related to the GP gene family. The gene is ~46 kb long and includes 15 exons, and polymorphisms have been identified at >32 bases in the human gene 13.

**Figure 1:**
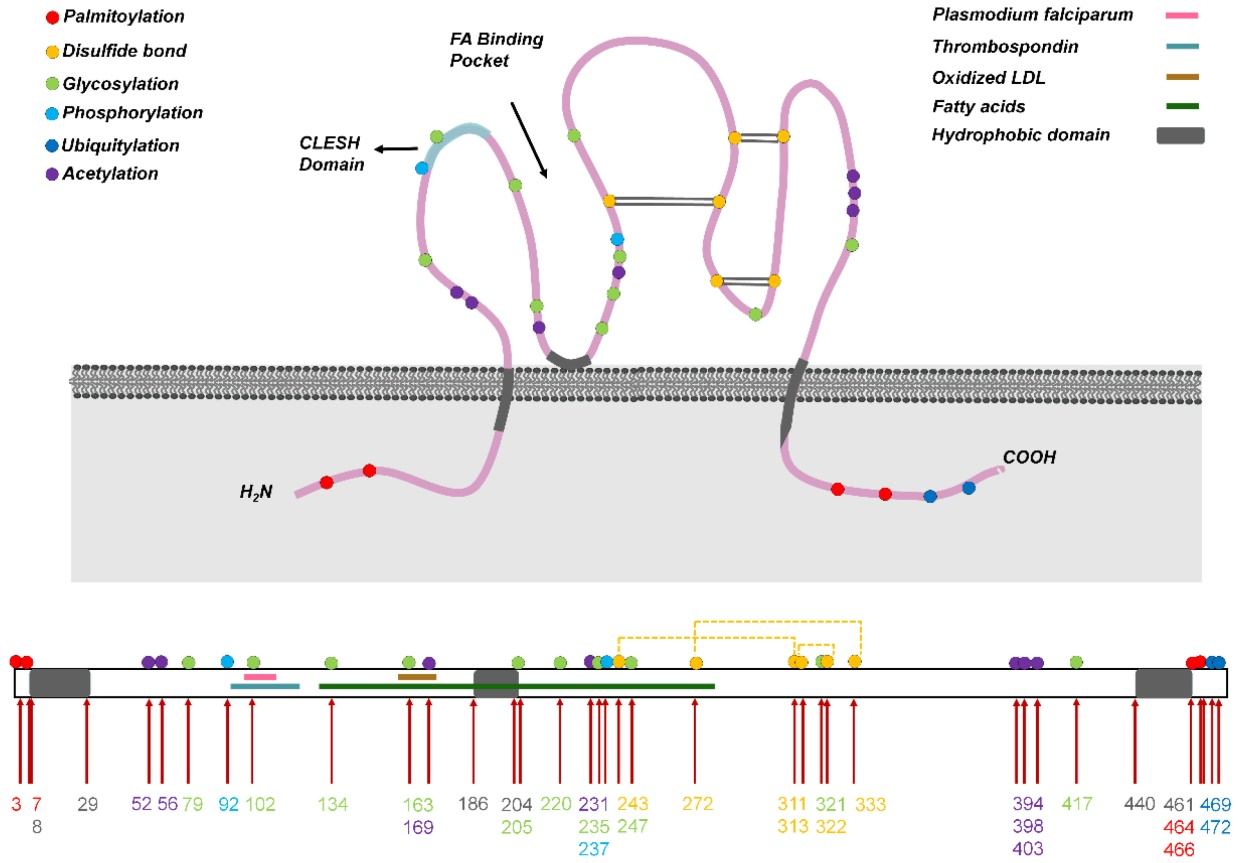
Structure of CD36. (Above) CD36 harbors two transmembrane domains, a large extracellular region containing ligand-binding sites, and one short cytoplasmic tail at each terminal (N and C). The extracellular domain of CD36 forms two hydrophobic cavities that bind to ligands such as fatty acids and oxidized LDL (ox-LDL). Moreover, the CLESH domain (CD36, LIMP-2, Emp sequence homologous domain) residues are negatively charged and interact with TSP-1 repeat domain 2 (TSR). CD36 undergoes multiple posttranslational modifications, including palmitoylation, acetylation, glycosylation, phosphorylation, ubiquitylation, and disulfide bonding (at three sites), and these modifications control CD36 maturation and localization in cells. Conversely, ligands bind to CD36 in different regions, and the binding leads to the activation of various downstream pathways. (Below) CD36 primary structure showing the hydrophobic domain and binding sites of general ligands. The numbers in different colors denote amino acid sites that undergo posttranslational modification: red, palmitoylation; green, glycosylation; blue, phosphorylation; dark blue, ubiquitylation; purple, acetylation.

Human CD36 contains ~472 amino acids and its estimated molecular weight is 53 kDa 10; the protein harbors two transmembrane domains, a large extracellular region containing ligand-binding sites, and a single short cytoplasmic tail at each terminal (N and C) (**Figure 1**). The extracellular domain of CD36 forms two hydrophobic cavities that mediate the uptake of hydrophobic molecules such as FAs, phospholipids, and cholesterol 13. Moreover, in CD36, the CD36, LIMP-2, Emp sequence homologous (CLESH) domain residues are negatively charged and can interact with TSP-1 repeat domain 2 (TSR) to regulate tumor angiogenesis, platelet aggregation, and other reactions, and CD36 also harbors a lysine-cluster region that can bind to negatively charged ligands 14. Almost all end-stage oxidative products are negatively charged, including ox-LDL, apoptotic cells, and advanced oxidation protein products (AOPPs) 15, which might be present at adequate levels in areas that exhibit heightened metabolism, such as in tumor tissues.

Besides the transmembrane form of CD36, a soluble form of the protein, sCD36, is also generated, and this form was shown to be composed of the exposed extracellular domain of transmembrane CD36 that is shed by plasma proteases or a specific subset of circulating microparticles 16. The mechanism underlying sCD36 generation warrants further investigation.

### CD36 distribution in cancer

In tumor tissues, CD36 is expressed in tumor cells 4, MVECs 17, stromal cells 5, and immune cells 18, and the CD36 level varies in distinct cell types. In malignant epidermal tumor cells, such as in the cells of ovarian cancer 19, gastric cancer 20, glioblastoma (GBM) 21, and oral squamous cell carcinoma (OSCC) 4, CD36 expression is markedly upregulated, whereas in tumor microvessels, which support tumor development and metastasis, CD36 expression is generally downregulated. CD36 expression is also deficient in the tumor stroma. For example, CD36 expression level in breast cancer stroma is considerably lower than that in adjacent tissues. Furthermore, a strong risk factor associated with high prevalence of breast cancer is high mammographic density (MD), and high-MD (but cancer-free) tissues were also shown to express reduced levels of CD36 5. These lines of evidence suggest that CD36 deficiency is representative of the tumor stroma and high cancer risk: the lower the CD36 level in the stroma, the more aggressive the tumor.

CD36 is widely recognized as a plasma membrane protein. On the cell membrane, CD36 and caveolin-1 can co-polymerize in plasma membrane domains called caveolae 22, which are specialized cell-membrane microdomains that are rich in cholesterol, sphingomyelin, signaling molecules, and receptor proteins and promote the integration of signaling cascades 23. CD36 can be transported to organelle and cell membranes by intracellular and extracellular vesicles and can regulate FA uptake and the balance of energy metabolism. CD36 transport to the cell membrane can be facilitated by several physiological stimuli, the most potent of which are insulin and muscle contraction. Muscle contraction induces CD36 localization in the cell membrane by activating adenosine 5ʹ-monophosphate-activated protein kinase (AMPK) 24, whereas insulin produces this effect by activating the phosphatidylinositol 3-kinase (PI3K)/AKT signaling axis 25. Moreover, inflammation promotes CD36 transcription, translation, and translocation to the cell surface 26.

CD36 is also present in endosomes, the endoplasmic reticulum (ER), and mitochondria. In the ER, CD36 undergoes modification and maturation as well as ubiquitylation-mediated inactivation. Furthermore, CD36 plays a critical role in mitochondrial FA oxidation (FAO) in skeletal muscle, and CD36 functions in regulating mitochondrial FAO together with carnitine palmitoyltransferase-1 (CPT1) 27. To elucidate CD36 function(s) in cancer, it is of interest to comprehensively investigate the regulation of CD36 expression and distribution.

## Regulation and localization of CD36

### Regulation of CD36 gene

**Figure 2:**
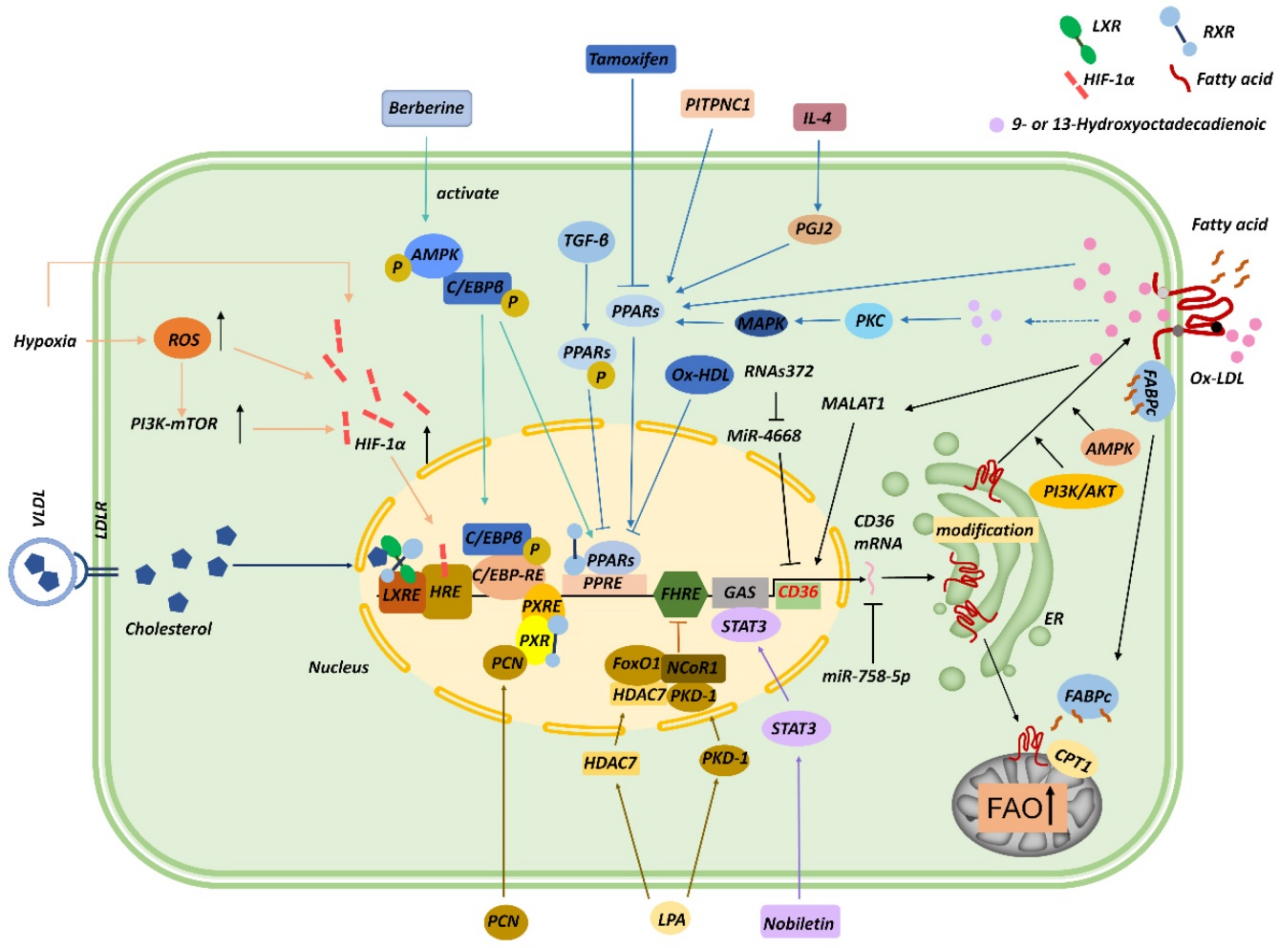
Regulation of CD36. CD36 transcription can be modulated by several transcription factors and their ligands, including PPARs, C/EBP, STAT3, LXR, PXR, FoxO1, and HIF-1α. Noncoding RNAs, including miR-758-5p, MALAT1, miR-4668, and RNAs372, can also regulate CD36 mRNA levels. CD36 is posttranslationally modified in the ER during maturation, and these posttranslational modifications and the AMPK and PI3K/AKT signaling pathways modulate the translocation of CD36 to the membrane. CD36 trafficked to mitochondria functions in cooperation with CPT1 to promote FAO.

Several transcription factors contribute to *CD36* transcription (**Figure 2**). For instance, the promoter of *CD36* contains peroxisome proliferator-activated receptor (PPAR)-response elements (PPREs) 28, and thus PPARγ ligands, including 9- and 13-hydroxyoctadecadienoic acid (HODE), ox-LDL, and prostaglandin J2 (PGJ2), upregulate *CD36* mRNA expression 29, 30. Moreover, interleukin (IL)-4 induces *CD36* expression in macrophages by activating intracellular lipoxygenase and the PPARγ ligand PGJ2 31. In gastric cancer, phosphatidylinositol transfer protein cytoplasmic 1 (PITPNC1) upregulates the RNA level of *PPARG*, and PPARγ then enhances the expression of *CD36* and mitochondria *CPT1* and thereby elevates FA absorption and promotes FAO and metastasis 32. Conversely, tamoxifen inhibits *CD36* expression and ox-LDL accumulation by inactivating PPARγ signaling 33, and *CD36* downregulation induced by transforming growth factor-β (TGF-β) was found to be associated with the phosphorylation and inactivation of PPARγ 34. In macrophages, ox-LDL and its metabolites 9-/13-HODE absorbed by CD36 activate PPAR through protein kinase C (PKC), protein kinase B (PKB), and p38 mitogen-activated protein kinase (MAPK) pathways 35. PPAR combines with retinoid X receptor (RXR) to form dimers at *CD36* promoter and enhances *CD36* expression, which, in turn, increases ox-LDL uptake; this signaling contributes to foam-cell formation 36, 37. By contrast, oxidized HDL (ox-HDL) inhibits *CD36* expression through a PPARγ-dependent mechanism in macrophages 38. Intriguingly, pregnane X receptor (PXR) reaction components, liver X receptor (LXR) nuclear receptor-binding site, and a CCAAT/enhancer-binding protein (C/EBP)-response element were also found in the promoter of *CD36* 37, and, interestingly, C/EBP can bind to PPREs and activate PPARγ 39.

Lysophosphatidic acid (LPA) is a phospholipid that stimulates cell proliferation 40. LPA can initiate the assembly of nuclear transcription complexes in the microvascular endothelium through protein kinase D (PKD1), which induces the recruitment of a co-repressor, nuclear receptor co-repressor 1 (NCoR1), and histone deacetylase 7 (HDAC7) to the forkhead box O1 (FoxO1) nuclear complex. FoxO1 inhibits *CD36* transcription, which leads to diminished cellular binding of TSP-1 17. This process might be related to the promotion of tumor angiogenesis. However, the opposite effect was observed in macrophages. Moreover, LPA and its analogs are agonists of PPARγ, which upregulates *CD36* expression and induces lipid accumulation through ox-LDL absorption. LPA can also be excreted from stimulated cancer cells. Thus, LPA derived from activated tumor cells might regulate *CD36* in adjacent or distant target cells 41.

Signal transducer and activator of transcription 3 (STAT3) can bind to the interferon-γ-activated sequence (GAS) element sequence (TTCCATGAA) in the *CD36* promoter region. Nobiletin, a flavonoid isolated from citrus peel, was shown to block STAT3 binding of the GAS element and *CD36* promoter and thereby inhibit angiogenesis, tumor invasion, and metastasis 42. Mwaikambo and colleagues recently reported that hypoxia upregulated *CD36* expression on retinal MVECs through the hypoxia inducible factor-1 (HIF-1) and PI3K pathways 43. Moreover, Notch signaling can be affected by plasma glucose and inflammatory lipids and is closely related to the metabolic status of cells; the Notch signal inhibits angiogenesis in vascular system development and in solid tumors, which is of important clinical significance in tumor therapy. Japs and colleagues found that in endothelial cells, *CD36* transcription was upregulated by molecules that function downstream of Notch binding to the Rbp-jκ-binding sites (TG[G/A]GAA) of *CD36* promoter 44. Furthermore, *CD36* expression at the transcriptional level is enhanced by macrophage colony-stimulating factor (M-CSF) 45, natural and modified LDLs 46, cellular cholesterol 47, glucose molecules 48, and IL-4 46, whereas *CD36* transcription is downregulated by interferon 49, glucocorticoids 50, TGF-β 34, tumor necrosis factor-α (TNF-α) 51, lipopolysaccharide (LPS) 50, and statins 52.

Recently, noncoding RNAs were found to regulate *CD36*. In foam cells derived from THP1 macrophages, miR-758-5p reduces cholesterol uptake and lipid accumulation in cells by downregulating *CD36* mRNA and protein levels 53. Moreover, ox-LDLs increase the transcription of the long noncoding RNA MALAT1 through the NF-κB pathway, which enriches the binding site for β-catenin on *CD36* promoter and increases the uptake of lipids in foam cells 54. MiR-4668 and miR-26a can bind to the 3ʹ noncoding region of *CD36* and inhibit *CD36* transcription 55, whereas highly-conserved RNAs372 promotes *CD36* transcription by blocking the maturation of miR-4668 56.

In lung cancer, increased methylation of *CD36* promotes cancer progression, whereas decitabine and chidamide synergistically inhibit the growth of lung tumor; these findings indicate that *CD36* demethylation might represent a potential strategy for tumor therapy 57.

### Regulation of CD36 protein

Very few previous studies have investigated the regulation of CD36 protein translation, which thus warrants increased research attention in the future. Notably, however, CD36 is known to contain multiple posttranslational modification sites that can be glycosylated, phosphorylated, palmitoylated, acetylated, or ubiquitylated, and these modifications regulate CD36 stability, protein folding, and translocation.

CD36 is glycosylated in the ER and Golgi, and this promotes CD36 structural stabilization and trafficking to the plasma membrane 58. CD36 harbors 10 potential glycosylation sites, all within the extracellular ring, and fully glycosylated CD36 is a transmembrane GP receptor of ~88 kDa. High-level glycosylation of CD36 promotes CD36 expression on the cell membrane without affecting its ligand binding 59. In intestinal epithelial cells, enhanced glycosylation of CD36 has been reported to increase FA uptake partially through an elevation of membrane CD36 levels 60.

CD36 contains two phosphorylation sites, Thr92 and Ser237, that can regulate extracellular ligand binding. CD36 Thr92 phosphorylation in melanoma cells is induced by phorbol-12-myristate-13-acetate (PMA) during protein synthesis and transport through the Golgi 61, and CD36 Ser237 phosphorylation inhibits FA uptake by platelets and intestinal cells. Because the two CD36 phosphorylation sites are adjacent to the CLESH domain of CD36, their phosphorylation spatially inhibits the binding of TSP-1 and CLESH domain and thereby regulates collagen adhesion. A previous study showed that endothelial CD36 is constitutively phosphorylated at Thr92, which maintains the adhesion of *Plasmodium falciparum*-infected erythrocytes to human dermal MVECs under flow conditions 62. Furthermore, in melanoma cells, CD36 Thr92 phosphorylation has been shown to reduce the recruitment of Src-family proteins after TSP-1 binding to CD36 and blunt vascular-cell signaling 61.

Palmitoylation regulates protein subcellular localization, membrane interaction, and subcellular transport, and protein palmitoylation level is positively correlated with the plasma level of palmitic acid (PA). CD36 contains four palmitoylation sites, Cys3, Cys7, Cys464, and Cys466, located in the N- and C-terminal intracellular segments. Under PA stimulation, palmitoyl acyltransferases (PATs) induce CD36 palmitoylation in the ER, which is a reversible process. Recently, two aspartate-histidine-histidine-cysteine (DHHC) motif-containing PATs, DHHC4 and DHHC5, were reported to control CD36 palmitoylation at different subcellular locations and affect CD36 plasma membrane translocation and FA-uptake activity 63. Palmitoylated CD36 is located in lipid rafts in the cell membrane and mediates FA adsorption and transport. Whereas palmitoylation increases the lipid-uptake activity of CD36, which promotes the downstream pathways of inflammation and metabolic reprogramming, depalmitoylation inhibits CD36 maturation. Notably, palmitoylation deficiency was found to reduce the half-life of CD36 64. Therefore, inhibition of CD36 palmitoylation might represent a potential strategy for reducing CD36-mediated lipid accumulation and inflammatory signaling. Patients with non-alcoholic steatohepatitis exhibit elevated palmitoylation of CD36, which aggravates lipid accumulation and immune-cell infiltration in the liver 65, and tumor cells exhibiting metastatic potential express increased levels of CD36 and show elevated FA-uptake activity. However, the effect of CD36 palmitoylation in tumor cells remains to be investigated.

Protein acetylation is a major mechanism for regulating various protein functions and properties, including DNA replication and transcriptional activity 66, protein stability, FA metabolism 67, subcellular localization 68, and apoptosis 69. CD36 contains four acetylation sites, Lys52, Lys166, Lys231, and Lys403, but little is known regarding their biological functions 67.

CD36 undergoes ubiquitylation—both polyubiquitylation and monoubiquitylation—at Lys469 and Lys472. Protein polyubiquitylation leads to protein degradation by directing the target proteins to proteasomes, and in the C2C12 mouse muscle cell line, the degree of polyubiquitylation was found to affect the total CD36 expression level rather than CD36 distribution 70. The C-terminal lysines in CD36 can also be monoubiquitylated by parkin, and this is considered to be nondegradative 71. Parkin-knockout mice show reduced hepatic CD36 levels and an attenuated maladaptive response to high-fat diet 72. However, because parkin can also trigger polyubiquitylation, parkin functions vary in different tissues 73.

CD36 can also be degraded through the autophagy-lysosome-dependent pathway. C1q/TNF-related protein 13 (CTRP13) is a soluble adipokine that can reduce inflammatory responses, with a decrease in macrophage content, in lesion areas. In patients with coronary artery diseases, CTRP13 is invariably downregulated, and CTRP13 overexpression reduces CD36 levels by attenuating autophagy-lysosome-dependent degradation at the posttranslational level, which decreases ox-LDL uptake and foam-cell formation 74.

In summary, CD36 can be regulated at the gene and protein levels. Elevated lipid deposition and inflammatory factors in tumor tissues might induce and alter CD36 expression and distribution, and this could contribute to a high metastatic rate. Another key factor of interest is the level of the CD36 posttranslational modifications that can regulate the binding of ligands.

## Various ligands of CD36

The functional diversity of CD36 depends on the binding of distinct ligands. CD36 ligands can be classified as lipid-related and protein-related ligands. As a lipid transaminase, CD36 can absorb lipid molecules, including LCFAs 75, ox-LDL 76, anionic phospholipids 77, and oxidized phospholipids (ox-PLs) 78. Moreover, cell-membrane CD36 can transfer FAs to cytosolic FA-binding protein (FABPc) and transport it to mitochondria to provide energy for cell metabolism. CD36 also binds to protein-related ligands such as TSP-1, TSP-2, amyloid proteins, AOPPs 79, and advanced glycation end products (AGEs) 15. TSP-1 can bind to the CLESH domain of CD36 expressed on MVECs, which then activates the downstream Src-family pathway and mediates the apoptosis of endothelial cells.

As a scavenger receptor, CD36 can bind to other transmembrane proteins on the membrane surface, such as integrins (1, 2, and 5) and four-transmembrane proteins (CD9, CD81), which jointly mediate ligand binding and signal transduction. In GBM stem cells, CD36 is co-expressed with integrin-6 and CD133, and a decrease in CD36 results in the loss of integrin-6 expression, self-renewal, and tumor initiation 21. Furthermore, by acting as a regulator of the assembly of toll-like receptor (TLR) 4 and 6 heterodimers, CD36 can trigger inflammatory signal transduction in microglia upon encountering exogenous stimuli and ox-LDL 80.

CD36 also functions in innate immunity as a pattern-recognition receptor (PRR) by recognizing exogenous materials such as red blood cells infected by *Plasmodium falciparum* and the cell-wall components of staphylococcus and mycobacterium 81. Moreover, CD36 recognizes endogenous ligands: CD36 on DCs can recognize apoptotic cells and cross-present antigens to T cells 82, and CD36 on macrophages can bind to β-amyloid peptides 83, apoptotic cells 84, AOPPs 85, and AGEs 86.

In tumor cells, CD36 associates with multiple ligands that influence the development of cancer. FA uptake by CD36 for FAO supports metastasis 87, whereas CD36 binding of TSP-1 inhibits tumor progression 88, and hepatoma cells absorb ox-LDL through CD36 and SR-BI 89. These ligands determine the biofunctions of CD36 in cancer.

## Functions of CD36 in cancer

In recent decades, in-depth research on CD36 in cancer has elucidated CD36 functions in tumor-cell metabolism, anti-angiogenesis, metastasis, therapy resistance, and tumor immunoediting.

### CD36 accelerates tumor growth

Tumor cells are now recognized to possess the ability for enhanced lipid deposition and metabolism to meet the requirements of rapid proliferation and development 90. As a scavenger receptor, CD36 exhibits high affinity to transport LCFAs, which contribute to tumor growth. For instance, in a cervical cancer xenograft model, a high oleic acid diet increases tumor volume and weight, and inhibition of CD36 prevents this effect. Furthermore, oleic acid facilitates tumor development by activating Src kinase and the downstream ERK1/2 pathway in a CD36-dependent manner 91. These findings suggest that targeting CD36 could serve as a potential strategy against tumor growth.

Because of the vigorous metabolism and high energy requirement of cancers, a high demand for nutrients and oxygen frequently exists in the tumor microenvironment. Previous work has shown that in the absence of the ability to promote endogenous growth of new blood vessels, tumor diameter cannot exceed 1-2 mm 92. Therefore, the formation and extension of blood vessels in tumor tissues are crucial for tumor initiation, development, and metastasis. TSP-1, which belongs to a family of structurally related proteins, is secreted by activated platelets, various epithelial cells, and mesenchymal cells. TSP-1 harbors a TSR sequence that can interact with the CLESH domain in CD36, as well as a potential TGF-β activation sequence that plays a critical role in tumor-cell metastasis 61.

**Figure 3:**
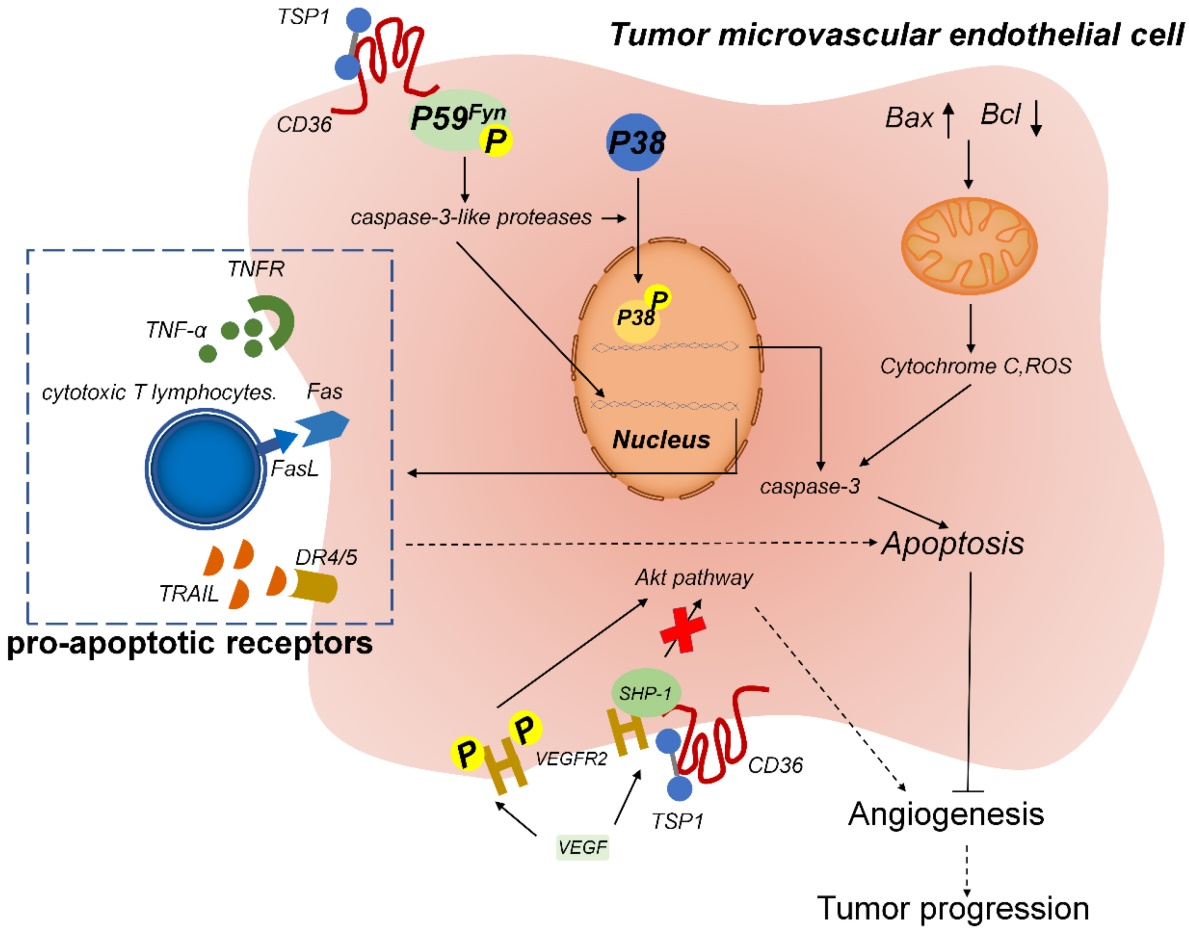
TSP-1-CD36 signaling induces apoptosis of tumor-associated endothelial cells. TSP-1 binds to CD36 on microvascular endothelial cells and induces the phosphorylation of the cytoplasmic protein tyrosine kinase P59*^fyn^*. Activated P59*^fyn^* stimulates caspase-3-like proteases, which activate and induce the phosphorylation of p38 mitogen-activated protein kinase (MAPK). Nuclear translocation of MAPK results in increased expression of caspase-3 and proapoptotic receptors, which leads to apoptosis. Furthermore, mitochondrial damage releases cytochrome C and reactive oxygen species (ROS), which also trigger the caspase-3 cascade to induce apoptosis. Moreover, TSP-1 biding to CD36 results in the recruitment of Src homology 2 domain-containing protein tyrosine phosphatase (SHP)-1 to the VEGFR2 complex and SHP-1-mediated dephosphorylation of VEGFR2, which inhibits the VEGF pathway and thus leads to anti-angiogenesis.

TSP-1, when bound to CD36 on the surface of MVECs, inhibits angiogenesis and promotes vascular apoptosis (**Figure 3**). On the MVEC surface, the binding of the membrane protein CD36 to TSR leads to the association of Fyn with the CD36 membrane complex, which increases the phosphorylation level of Fyn and then triggers p38 activation by caspase-3. Caspase-3 forms a proapoptotic complex and induces the expression of endogenous proapoptotic receptors, such as TNF receptor (TNFR), FAS, and the TRAIL (TNF-related apoptosis-inducing ligand) receptors death receptor (DR) 4 and DR5, which induce the apoptosis of endothelial cells. TSR treatment can reduce tumor growth and microvascular density in isogenic models of malignant melanoma and also in situ in models of human bladder cancer 93. Accordingly, recombinant TSP-2 fragments can inhibit angiogenesis and tumor growth in non-glioma tumors 94.

TSP-1 was recently found to inhibit vascular endothelial growth factor (VEGF) receptor 2 (VEGFR2) level through the CD36 pathway. In the microvascular endothelium, after CD36-TSP-1 binding, Src homology 2 domain-containing protein tyrosine phosphatase 1 (SHP-1) is recruited to the VEGFR2 signaling complex, and SHP-1 dephosphorylates this complex and attenuates VEGF signal transduction 95. TSP-1 and VEGF engage in an antagonistic relationship and jointly regulate the formation of microvessels. Thus, the CD36/TSP-1 anti-angiogenic signal switches the growth factor-mediated pro-angiogenic response to an anti-angiogenic and pro-apoptotic response 96.

In accordance with the aforementioned finding, TSR binds to CD36 on GBM cells and upregulates DR4/5 expression. Subsequently, signaling by DR4/5 initiates GBM apoptosis through TRAIL-induced expression of caspase-8/3/7 88. Together, these results suggest that TSR plays an antitumor role in tumor cells and tumor-related vascular endothelial cells, which reflects the considerable potential TSR holds for use in cancer therapy.

Histidine-rich glycoprotein (HRG) regulates microvascular CD36-TSR signaling; HRG is a 75-kDa protein synthesized by hepatocytes and circulated in plasma at high concentrations. Platelet α-particles are rich in HRG (109-371 ng/platelets), which can be released into specific microenvironments after platelet activation. HRG is a modular protein that can bind to proteoglycans, matrix proteins, divalent cations, and coagulin, and HRG contains a domain that is similar to the CLESH domain of CD36, which can bind to the TSRs of TSP-1/2 and angiostatin. Therefore, HRG, as a soluble decoy receptor of TSP-1/2, blocks the binding of CD36 to TSP-1 and inhibits the anti-angiogenic activity of TSP-1. In an experimental model in which mice were transplanted with Lewis lung-cancer and B16F1 melanoma cells, tumor volume was found to be markedly lower in the HRG-knockout group than in wild-type and CD36-knockout groups, and vascular density was also substantially lower in the HRG-knockout group than in CD36-knockout and wild-type groups 97.

Thus, TSP-1 and TSR associate with CD36 and trigger MVEC apoptosis and block VEGF signaling and thereby inhibit tumor growth. Consequently, targeting this pathway might provide a potential strategy for cancer therapy.

### CD36 promotes tumor metastasis

Tumor metastasis-initiating cells (MICs) represent a rare and unique population of cells derived from primary tumors that can seed metastasis colonies in secondary organs 98. GBM stem cells expressing high levels of CD36 were found to enhance self-renewal and tumor initiation 21. In 2016, Pascual reported that in human OSCC, CD44^+^ cell subsets highly express CD36 and lipid-metabolism genes and exhibit the unique ability to initiate metastasis 4. CD36-induced metastasis potential has been reported to be based on the lipid metabolism of cancer cells 99. Intriguingly, blockade of CD36 in OSCC cells also causes severe intercellular lipid accumulation in cancer cells, which leads to lipotoxic cell death and impaired metastasis 4.

CD36 mediates the uptake of FAs, which are key nutrients for tumor metabolism. PA uptake by CD36 has been shown to cause AKT phosphorylation in gastric cancer cells and inhibit glycogen synthase kinase 3β (GSK-3)/β-catenin degradation and promote gastric cancer metastasis 20. Tumor-associated adipocytes can provide sufficient FAs to tumor cells and thus promote their proliferation and metastasis. For example, gastric cancer commonly metastasizes to the greater omentum, which is rich in fat cells, and adipocytes also induce CD36 expression in metastatic and primary human ovarian tumors 100. In FAO, the rate-limiting step is FA transport into mitochondria, a process in which CPT1 and CPT2 are the key enzymes. CD36 present on mitochondria in human skeletal muscle cells can bind to CPT1, and CD36 upregulation in mitochondria is correlated with increased FAO. Notably, patients with myeloid leukemia who express elevated levels of CD36 also exhibit increased FAO, enhanced expression of oxidative phosphorylation (OXPHOS) and reactive oxygen species (ROS)-related genes, and poor prognosis 101.

CD36 also regulates FAO through the liver kinase B1 (LKB1)-AMPK pathway. AMPK regulates metabolic balance in cells, and this activates the uptake and metabolism of FAs and glucose. CD36 functions as an energy sensor of extracellular FA levels. When the FA level is low, CD36 forms a complex with Fyn and remains inactive, but when PA binds to CD36, Fyn dissociates from the CD36 intracellular domain and fails to phosphorylate LKB1; subsequently, elevated intracytoplasmic LKB1 phosphorylates and activates AMPK, which activates CPT1 and upregulates FAO by inhibiting acetyl-CoA carboxylase (ACC) and reducing malonyl-CoA content 102. Moreover, AMPK promotes CD36 transport to and localization at the cell membrane 22. For instance, in acute monocytic leukemia, bone marrow adipocytes can promote the expression of CD36 and AMPK genes in cancer cells, which increases FAO and mediates the survival and development of cancer cells 103.

Epithelial-mesenchymal transition (EMT) promotes cancer-cell metastasis 104. Aritro and colleagues found that CD36 uptake of FA led to EMT through Wnt and TGF-β signaling pathways 105. In the case of diabetic nephropathy, CD36 knockdown in HK-2 cells inhibited high glucose (HG)-induced ROS generation and TGF-β1 expression, which prevented HG-induced EMT in these cells 106. An analysis of The Cancer Genome Atlas revealed that increased *CD36* expression was consistently associated with high EMT in all cancers 107, although Pascual and colleagues found that CD36^+^ MICs expressed lower levels of EMT genes as compared with CD36^-^ MICs, which indicated that EMT might not participate in the metastasis initiation of all tumor types 108.

Taken together, these findings suggest that MICs and the cancer EMT process are both regulated by lipid metabolism through the CD36-dependent pathway. Therefore, CD36 might represent a potential target for the prevention and therapy of cancer metastasis.

As discussed above, TSP-1 has long been accepted to exert antitumor effects in various cancers; however, glioma cells overexpressing a TSP-1 fragment were found to be more aggressive and to decrease survival in animals as compared with glioma cells not expressing the fragment 109. These results suggest that although the TSP-1 fragment plays an anti-angiogenic role, it might also perform a cancer-promoting function. TSP-1 has been reported to promote metastatic spread by enhancing tumor-cell embolus formation and facilitating cancer-cell adhesion during metastasis 110. Furthermore, TSP-1 can release activated TGF-β by interacting with its potential precursors through RFK and WXXW sequences 111. TGF-β induces matrix production and alters integrin expression and thus promotes tumor growth by stabilizing angiogenesis 112, and activated TGF-β also upregulates urokinase (plasminogen activator), urokinase receptor, and plasminogen activator inhibitor-1 (PAI-1) in the proteolytic system and enhances the invasiveness of malignant tumors 113. Therefore, the TSP-1 effect on malignant metastasis partially depends on the TGF-β concentration at the tumor site in specific tumors. The pro-angiogenic effect of TGF-β and the anti-angiogenic effect of TSP-1 overexpression counterbalance each other, and this hinders the use of TSP-1 as an anti-angiogenesis drug in tumor treatment, a matter that warrants further investigation.

### CD36 regulates chemoresistance and radioresistance

Resistance to radiotherapy and chemotherapy poses a major challenge in cancer treatment. Tumor stem cells have been widely reported to play a crucial role in chemotherapy resistance, and metabolic alterations in these cells might be the key to their ability to maintain stemness and drug resistance. In acute myeloid leukemia cells that are resistant to arabinocytosine (AraC, also known as cytarabine), FAO and OXPHOS are elevated and CD36 is upregulated. Notably, reducing FAO, mitochondrial protein synthesis, and electron transfer maintains OXPHOS at a low level, and this can markedly enhance the anti-leukemia effect of AraC 101. In breast cancer, breast adipocytes provide adequate FAs for breast cancer stem cells (BCSCs). Whereas JAK/STAT3 signaling regulates lipid metabolism to promote FAO in BCSCs, which maintains the stemness and chemotherapy resistance of breast cancer cells, inhibiting FAO sensitizes breast cancer cells to chemotherapy 114. Because STAT3 can increase CD36 expression at the transcriptional level, these results suggest that CD36 could play a critical role in the chemoresistance of these cells. Moreover, as a result of their unique metabolic characteristics, CD36^+^ leukemic stem cells accumulate in adipose tissues; this allows the cells to be protected by the gonadal adipose tissue microenvironment and thereby escape the effects of chemotherapy 115.

In addition to killing tumor cells, radiotherapy inevitably causes damage to normal tissues and thus affects the overall therapeutic effect. A previous study showed that CD47 interaction with TSP-1 on the cell surface attenuated the post-treatment recovery ability of normal cells, and that normal tissues of mice showed enhanced resistance to radiation damage when the CD47-TSP-1 interaction was blocked 116. Because CD47 and CD36 can competitively bind to TSP-1, CD36 might endow tumor-radiotherapy resistance to normal tissues and protect nontumor tissues against radiation damage and enhance the efficacy of radiotherapy.

### CD36 modulates tumor immunity

**Figure 4:**
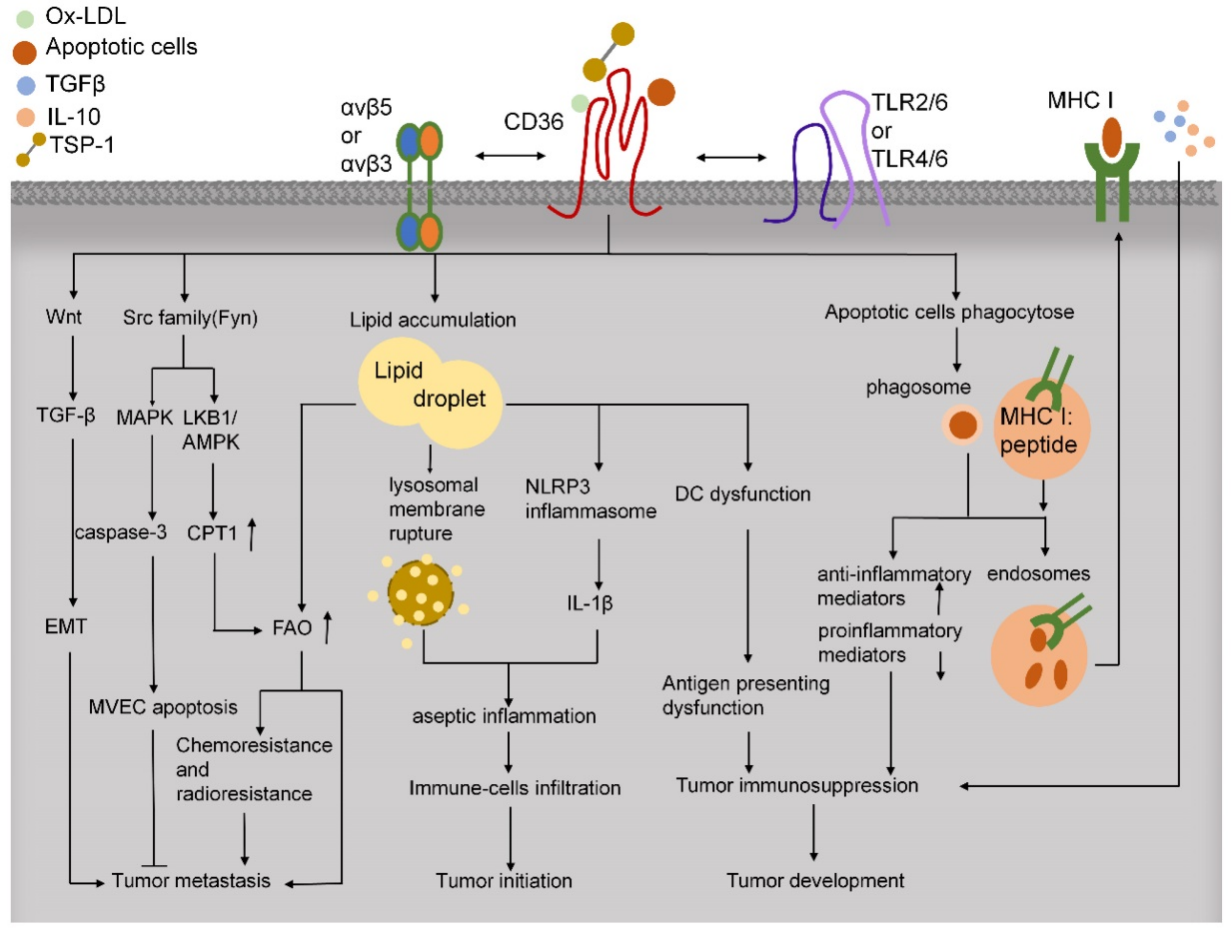
CD36 functions in tumor microenvironment. CD36 regulates downstream Src-family kinases to promote anti-angiogenesis, FAO, and chemoresistance and radioresistance, which lead to tumor metastasis. CD36 also activates Wnt/TGF-β signaling to facilitate tumor metastasis through EMT. CD36 uptakes multiple lipids, such as ox-LDL, LCFAs, and cholesterol. The lipid deposition in immune cells leads to aseptic inflammation and dysfunction of antigen presentation in DCs, which induce tumor immunosuppression. Moreover, CD36 can bind to apoptotic cells and activate cross-priming, which might lead to immunosuppression and tumor development.

In tumor tissues, immune cells play critical roles in facilitating tumor development and progression (**Figure 4**). CD36 regulates cytokine production, antigen presentation, phagocytosis, and immune tolerance. In macrophages, CD36 and TLRs function in the innate immune response by recognizing and engulfing pathogens and pathogen-associated molecules, including LPS from various gram-negative and gram-positive bacteria 117. CD36 can activate TLR4 and TLR6 to promote the release of inflammatory cytokines 80. Moreover, CD36-mediated internalization of ox-LDL leads to lysosomal membrane rupture and activation of NOD-like receptor protein-3 (NLRP3) inflammasome and thereby triggers sterile inflammation 118. CD36 deletion can alleviate the aseptic hepatitis caused by concanavalin A, suppress the infiltration of CD4+ and CD8+ T cells and natural killer (NK) cells, downregulate the levels of p-JNK, p-IKK, and cleaved caspase-3, and reduce the expression of various inflammatory mediators, including TNF-α, C-X-C motif ligand 10 (CXCL10), IL-1, monocyte chemoattractant protein-1 (MCP-1), and IL-6 119. Because inflammation triggers the initiation, proliferation, invasion, and metastasis of tumor cells, reducing CD36-mediated sterile inflammation might provide the basis for a new mode of antitumor treatment.

Lipid deposition causes dysfunction of antigen presentation by DCs and T-cell activation, and this promotes tumor immune tolerance and progression. As compared with DCs from tumor-free mice and healthy people, the DCs of tumor-bearing mice and cancer patients have been reported to contain a considerably larger proportion of triglycerides 120. CD36 is known to play an essential role in lipid uptake, but further investigation is required to elucidate the function of CD36 in lipid deposition in DCs.

In addition to internalizing oxidized lipids, phagocytes and antigen-presenting cells recognize anionic phosphatidylserine (PS) on the surface of apoptotic cells through CD36 121. In conjunction with αvβ5 integrin, CD36 recognizes and binds to specific epitopes on the surface of apoptotic cells, and activates CD8+ T cells through cross-priming and thus mediates the killing of malignant or infected cells 122. Therefore, reduced CD36 expression might help tumor cells in evading the immune system. Macrophages do not express αvβ5 integrin on the surface, and their surface CD36, in coordination with the entire αvβ3 integrin protein, phagocytoses apoptotic cells, but does not mediate antigen presentation.

In the tumor microenvironment, rapid proliferation of tumor cells leads to a lack of energy supply in the tumor-tissue core, which results in the formation of necrotic foci; consequently, a large number of apoptotic and necrotic cells can be consumed by tumor-associated macrophages (TAMs) and tumor-associated DCs in a CD36-denpendent manner. Because the phagocytosis of DCs and the presentation of apoptotic bodies can affect the TCR library of Treg and Tconv cells 121, tumor-associated DC phagocytosis might represent one of the mechanisms leading to tumor immune tolerance. Moreover, incubation of macrophages with apoptotic cells was found to increase the release of anti-inflammatory mediators and decrease the secretion of LPS-induced proinflammatory mediators, and therefore the apoptosis of these cells did not elicit a strong inflammatory response 123. Whether this mechanism mediates the development of the immunosuppressive environment in tumor tissues should be of future research interest.

CD36 is expressed during the late period of differentiation in tissue-resident macrophages and circulating and bone marrow-derived monocytes 124. Macrophages can polarize toward both M1 (classical) activation and M2 (alternative) activation 125. CD36-mediated uptake of triacylglycerol substrates and their subsequent lipolysis by lysosomal acid lipase (LAL) was found to promote M2 activation of macrophages, which then exhibited elevated OXPHOS and enhanced spare respiratory capacity (SRC) 126. Macrophage polarization toward M2 activation can also be induced by ox-LDL 127, but ox-LDL uptake through CD36 in eosinophils was found to promote the release of proinflammatory cytokines, which facilitated macrophage M1 polarization 128. Notably, CD36 expressed in macrophages plays a critical role in atherosclerosis 129. Blood-circulating monocytes transmigrate into the arterial intima, where the cells differentiate into macrophages and internalize ox-LDL through CD36 76. Moreover, internalized ox-LDL activates PPAR-γ, which, in turn, upregulates CD36 expression. Further uptake of excess ox-LDL contributes to cholesterol deposition in macrophages and generates foam cells, which is the initial critical step of atherosclerosis 29 130. The interaction of ox-LDL and CD36 also induces cytokine release and immune-cell infiltration, which accelerate the process of atherosclerosis 131.

TAMs are a group of tumor-infiltrating immune cells that are notable for their tumor-promoting ability 132. In breast cancer, apoptotic tumor cells release miR-375 and bind LDL, and this mediates their uptake by TAMs through CD36. Apoptotic tumor cells also release LCFAs and ox-PLs and thus act as carriers of miR-375 for CD36-mediated uptake, and miR-375, in turn, enhances TAM migration and infiltration into tumor spheroids. Moreover, in tumor cells, miR-375 increases CCL2 expression and thereby enhances the recruitment of macrophages, and knocking down CD36 largely prevents miR-375 uptake into macrophages 133. Thus, CD36 facilitates the migration and recruitment of TAMs in tumor tissues, which promotes tumor progression.

Metastasis-associated macrophages (MAMs) function in tumor metastasis by potentiating extravasation and enhancing the survival of metastasizing cancer cells. MAM precursor cells are found in metastasis sites at an early stage and accumulate during metastatic tumor growth 134. Distinct from normal circulating classical monocytes, MAMs harbor specific markers such as CD36 that contribute toward suppressing the cytotoxicity of activated CD8+ T cells 18. In this regard, CD36 might serve as a marker of a subset of MAMs.

Tumor microvesicles are a unique type of extracellular microvesicles that circulate in the peripheral blood of patients with metastatic cancer and contribute to distant metastasis. CD36 expressed on specific tissue immune cells bind and engulf tumor microvesicles, which leads to the invasion and extravasation of microvesicles from vessel walls and promotes metastasis 135. Therefore, targeting CD36 could represent a potential immunotherapeutic strategy for cancer.

## Clinical trials targeting CD36 for cancer treatment

As discussed in preceding sections, CD36 is closely related to tumor initiation, development, invasion, and metastasis, and thus numerous preclinical studies and clinical trials focused on CD36 have been and are being performed (**Table 1**). Because CD36 mediates FA uptake on the surface of tumor cells and is related to the metastatic potential of tumor cells, inhibiting CD36-mediated FA uptake on the tumor-cell surface could serve as a strategy for treating metastatic tumors. For example, FA uptake was found to be enhanced in human prostate cancer, and deleting CD36 in the prostate of cancer-susceptible *Pten*^-/-^ mice was reported to markedly reduce FAs uptake, which decreased the abundance of oncogenic signaling lipids and attenuated cancer progression 136. These findings support the conclusion that CD36-mediated FA uptake is critical for prostate cancer development, and targeting FA uptake might therefore represent an effective strategy for treating prostate cancer. Moreover, synthetic amphipathic helical peptides (SAHPs) that replicate the apolipoprotein domain were found to be capable of substituting for the binding of apolipoprotein to CD36; ELK-SAHPs effectively inhibited pulmonary inflammation and dysfunction in a sepsis model by inhibiting the FA-transport activity of CD36 137. Although the SAHPs clearly inhibited CD36 in the kidney and lung, their functional effect in cancer and in other tissues remains unclear.

CD36 expressed in the tumor microvascular endothelium binds to TSP-1 expressed in tumor tissues and mediates the apoptosis of tumor vascular endothelial cells. Cancer-treatment studies targeting TSP-1 binding to CD36 have yielded promising results 138. Three modified TSR peptides, ABT-526, ABT-510, and ABT-898, were all found to function effectively in animal experiments, and ABT-510 even reached phase 2 clinical trials. However, ABT-510 failed in phase 2 clinical trials because of ineffective performance and severe adverse events 139. Pfizer developed CVX-22 and CVX-045, which contain TSP-1-derived peptidomimetics and an antibody scaffold: CVX-045 caused potent regression of tumor xenografts by reducing tumor microvasculature and increasing necrotic cores, but it failed in a phase 1 clinical trial because of severe side effects and unsatisfactory efficacy 140; like CVX-045, CVX-22 was also found to produce beneficial effects in the preclinical phase, and thus this compound might serve as a potential CD36-targeting drug in the future 141.

CD47 acts as a “don't eat me” signal to macrophages. TSP-1 interacts with CD47 on immune cells and thereby inactivates antitumor immunosurveillance, and, concomitantly, attenuates the antitumor effect by interacting with CD36. Recently, a cyclic peptide named TAX2, derived from CD47, was discovered to bind TSP-1 directly and antagonize TSP-1/CD47 interaction, which inhibited angiogenesis through the CD36-dependent pathway. More intriguingly, the effects of TAX2 appeared to be restricted to the tumor-associated environment in which TSP-1 was overexpressed 142. TAX2 induced a switch in TSP-1 binding from CD47 to CD36, which led to an anti-angiogenesis effect in tumors. TAX2 reduced the tumor volume in allogeneic melanoma and effectively inhibited xenograft growth in pancreatic cancer, as well as caused the destruction of tumor-related vascular networks 143. In future work, TAX2 use might offer a new strategy for tumor anti-angiogenesis therapy.

## Conclusion and Perspectives

CD36 is a multifunctional molecule involved in several diseases, including cancer, metabolic diseases, Alzheimer's disease, and infection. CD36 plays a critical role in lipid uptake, immunological recognition, inflammation, molecular adhesion, and apoptosis, which influence the initiation, development, and progression of cancer. The regulation and localization of CD36 are controlled by complex signaling pathways, which might provide potential drug targets for tumor therapy. CD36 posttranslational modifications, including palmitoylation, phosphorylation, and glycosylation, modulate the translocation and ligand binding of CD36, and this could also potentially offer promising targets for regulating tumor-cell metabolism. Diverse ligands and pathways lead to tumor anti-angiogenesis, metastasis, immunity, and therapy resistance through CD36. CD36 interacts with TSP-1 through the binding of the CLESH domain and TSR, and this contributes to MVEC apoptosis and inhibits VEGF signaling and thereby leads to anti-angiogenesis in tumor tissues. Although TSP-1 might also promote tumor progression through TGF-β in certain tumor types, drugs targeting the TSP-1-CD36 anti-angiogenic effect perform favorably and have already entered phase 2 clinical trials. Moreover, CD36 was also identified to function as the enzyme FAT in the uptake of LCFAs, ox-LDLs, PS, and cholesterol, which promote tumor metastasis. High-level expression of CD36 in tumor cells not only facilitates FAO to supply adequate energy for tumor progression and chemoresistance, but also promotes EMT to enhance tumor-cell migration. Because ablation of CD36-mediated FA uptake attenuates tumor progression, targeting FA uptake by CD36 could represent an effective strategy for tumor treatment. As a scavenger receptor, CD36 plays a vital role in innate immunity. CD36 promotes sterile inflammation and the protumor ability of tumor-associated immune cells, which could serve as a potential target for tumor immunotherapy.

Several antitumor drugs targeting CD36 have already entered clinical trials, but the majority of these have failed because of severe adverse events and unsatisfactory performance. Thus, it is necessary to further investigate the regulation and downstream signaling pathways of CD36 during the manipulation of this target in precise clinical translation in the future.

## Figures and Tables

**Table 1:**
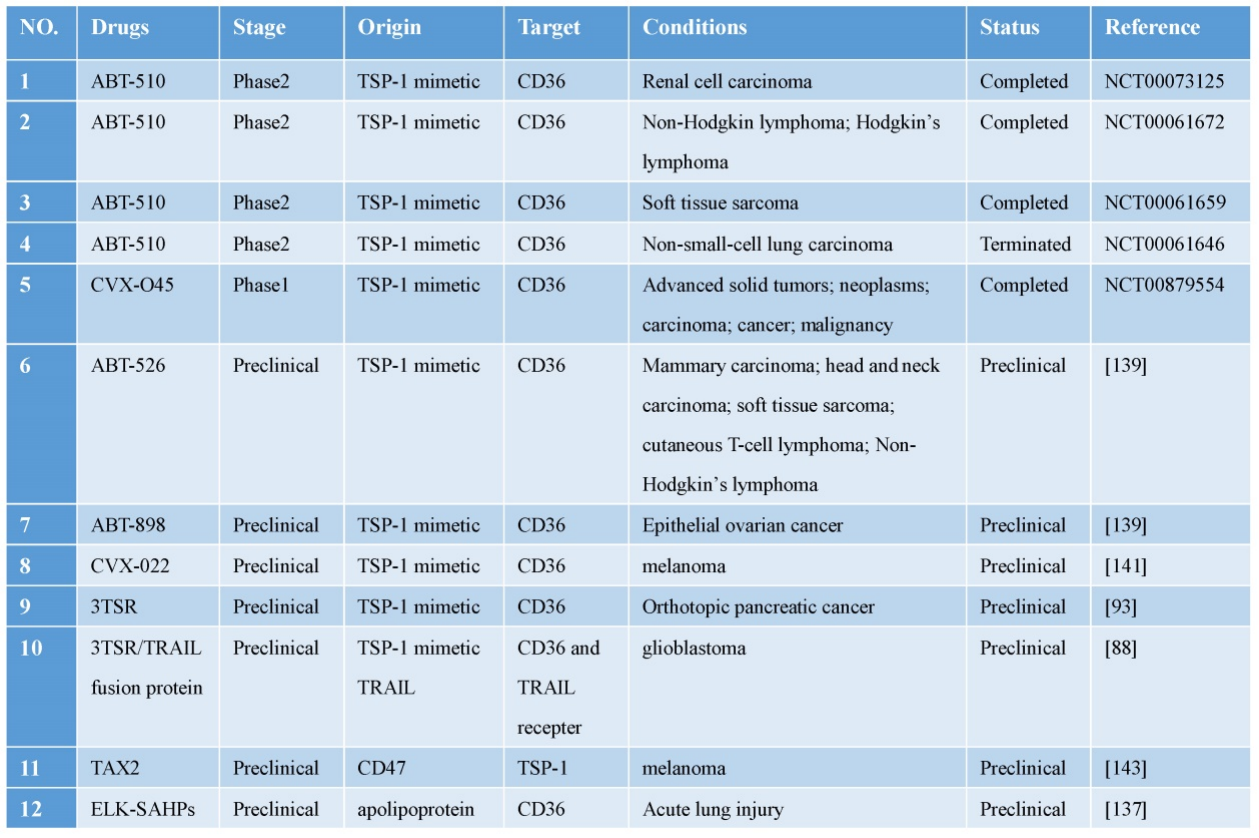
Clinical trials and preclinical drugs targeting CD36 for antitumor treatment.

Abbreviations: TRAIL, TNF-related apoptosis-inducing ligand; TSP, thrombospondin; TSR, TSP-1 repeat domain 2.

## Abbreviations

ACC: acetyl-CoA carboxylase
AGEs: advanced glycation end products
AMPK: adenosine 5'-monophosphate-activated protein kinase
AOPPs: advanced oxidation protein products
AraC: arabinocytosine
BCSC: breast cancer stem cells
C/EBP: CCAAT/enhancer-binding protein
CLESH: CD36, LIMP-2, Emp Sequence Homologous domain
CPT-1: carnitine palmitoyltransferase-1
CTRP13: C1q/tumor necrosis factor-related protein 13
CXCL10: C-X-C motif ligand 10
DCs: dendritic cells
DHHC: aspartate-histidine-histidine-cysteine
DR: death receptor
EMT: epithelial-mesenchymal transition
ER: endoplasmic reticulum
FABPc: cytosolic fatty acid binding protein
FAO: fatty acid oxidation
FAs: fatty acids
FAT: fatty acid translocase
FoxO1: forkhead box O1
GAS: interferon-gamma activated sequence
GBM: glioblastoma
GM-CSF: granulocyte-macrophage colony-stimulating factor
GP: glycoprotein
GSK-3: glycogen synthase kinase 3β
HDAC7: histone deacetylase 7
HG: high glucose
HIF-1: hypoxia inducible factor 1
HODE: hydroxyoctadecadienoic acid
HRG: histidine-rich glycoprotein
IL4: interleukin-4
IRBCs: *plasmodium falciparum*-infected erythrocytes
LAL: lysosomal acid lipase
LCFAs: long chain fatty acids
LPA: lysophosphatidic acid
LPS: lipopolysaccharide
LXR: liver X receptor
MAM: metastasis-associated macrophages
MAPK: mitogen-activated protein kinase
MCP-1: monocyte chemoattractant protein-1
M-CSF: macrophage colony-stimulating factor
MD: mammographic density
MICs: metastasis-initiating cells
MVECs: microvascular endothelial cells
NCoR1: nuclear receptor co-repressor 1
NK cells: natural killer cells
NLRP3: NOD-like receptor protein-3
OSCC: oral squamous cell carcinomas
ox-HDL: oxidized HDL
ox-LDL: oxidized low-density lipoprotein
OXPHOS: oxidative phosphorylation
ox-PLs: oxidized phospholipids
PA: palmitic acid
PAI-1: plasminogen activator inhibitor 1
PATs: palmitoyl-acyl transferases
PGJ2: prostaglandin J2
PI3K: phosphatidylinositol 3-kinase
PITPNC1: phosphatidylinositol transfer protein, cytoplasmic 1
PKB: protein kinase B
PKC: protein kinase C
PKD1: protein kinase D
PMA: phorbol-12-myristate-13-acetate
PPAR: peroxisome proliferator-activated receptor
PPREs: peroxisome proliferator-activated receptor response elements
PRR: pattern recognition receptor
PS: phosphatidylserine
PXR: pregnane X receptor
ROS: reactive oxygen species
RXR: retinoid X receptor
SAHPs: synthetic amphipathic helical peptides
SHP-1: Src homology 2 domain-containing protein tyrosine phosphatase 1
SRC: spare respiratory capacity
STAT3: signal transducer and activator of transcription 3
TAMs: tumor-associated macrophages
TGF-β: transforming growth factor-beta
TLR: toll-like receptor
TNFR: tumor necrosis factor receptor
TNFα: tumor necrosis factor-alpha
TRAIL: TNF-related apoptosis-inducing ligand
TSP-1: thrombospondin-1
TSR: TSP-1 repeat domain 2
VEGF: vascular endothelial growth factor
VEGFR2: vascular endothelial growth factor receptor 2
